# Supplementary figures and images for: Effectiveness of Schroth exercises for adolescent idiopathic scoliosis: a meta-analysis
Source: PeerJ. 2025 Jul 8;13:e19639. doi: 10.7717/peerj.19639 (PMC12248227; doi:10.7717/peerj.19639)

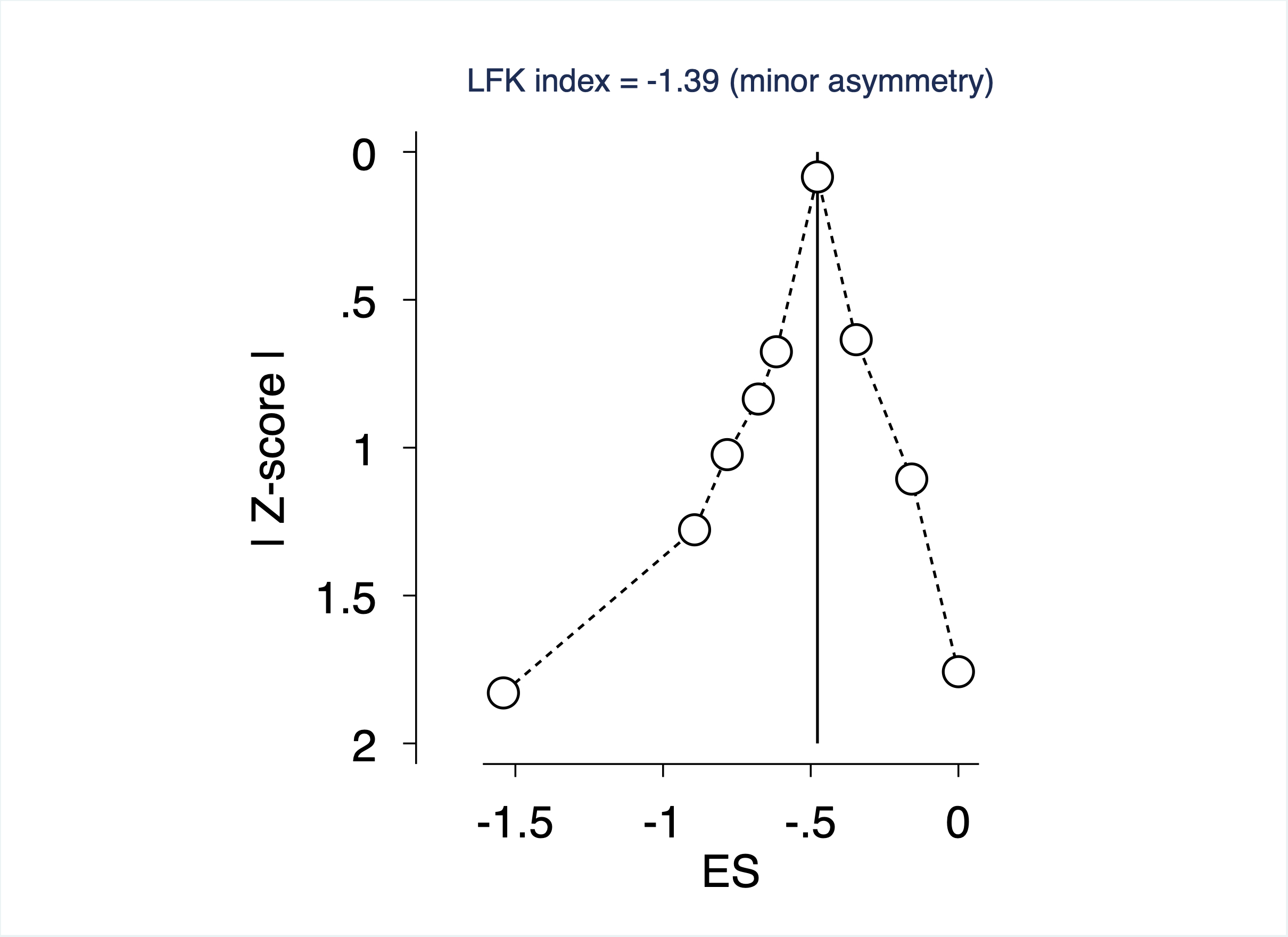

Supplement: Supplemental Information 2 [file peerj-13-19639-s002.tif]

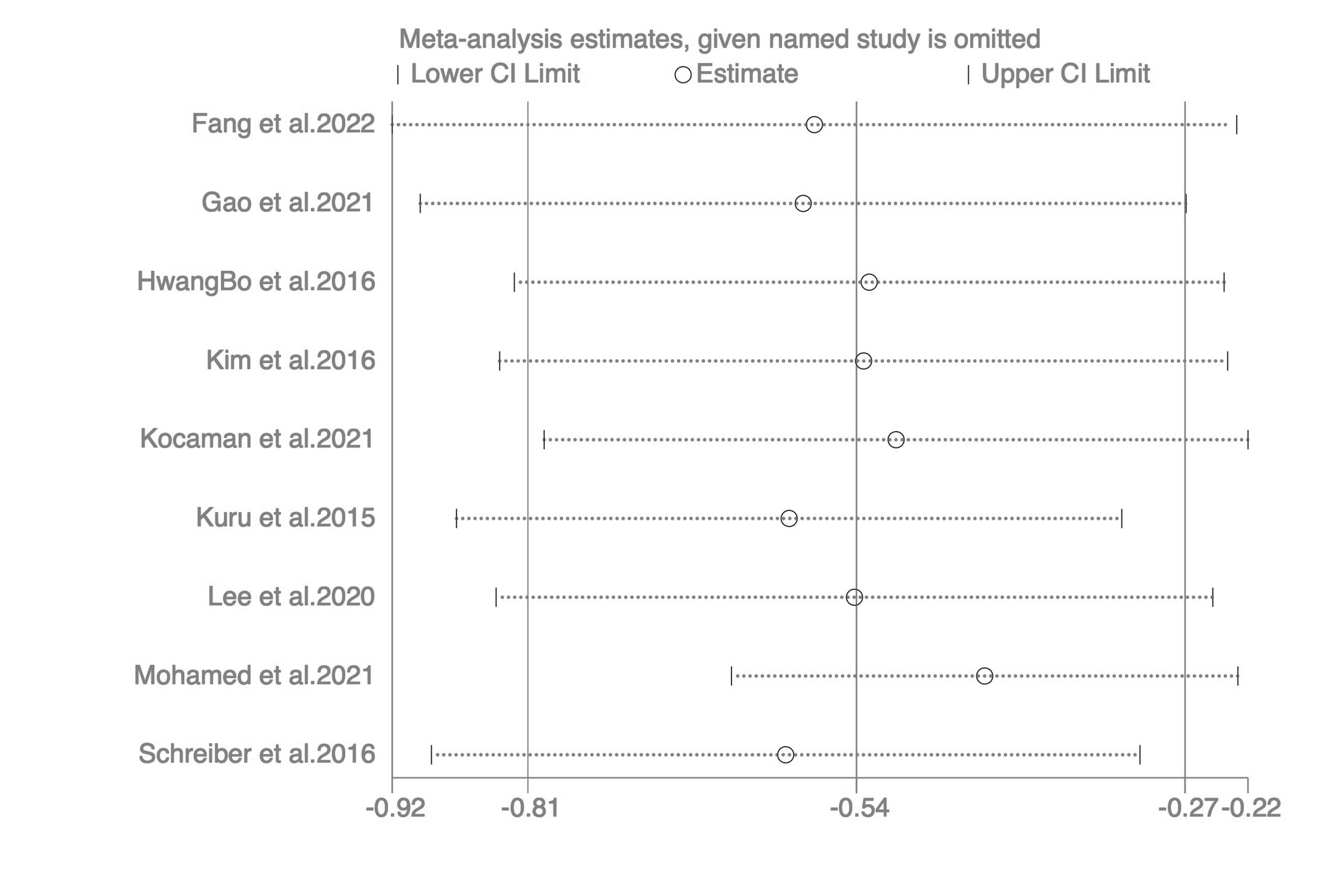

Supplement: Supplemental Information 3 [file peerj-13-19639-s003.tif]

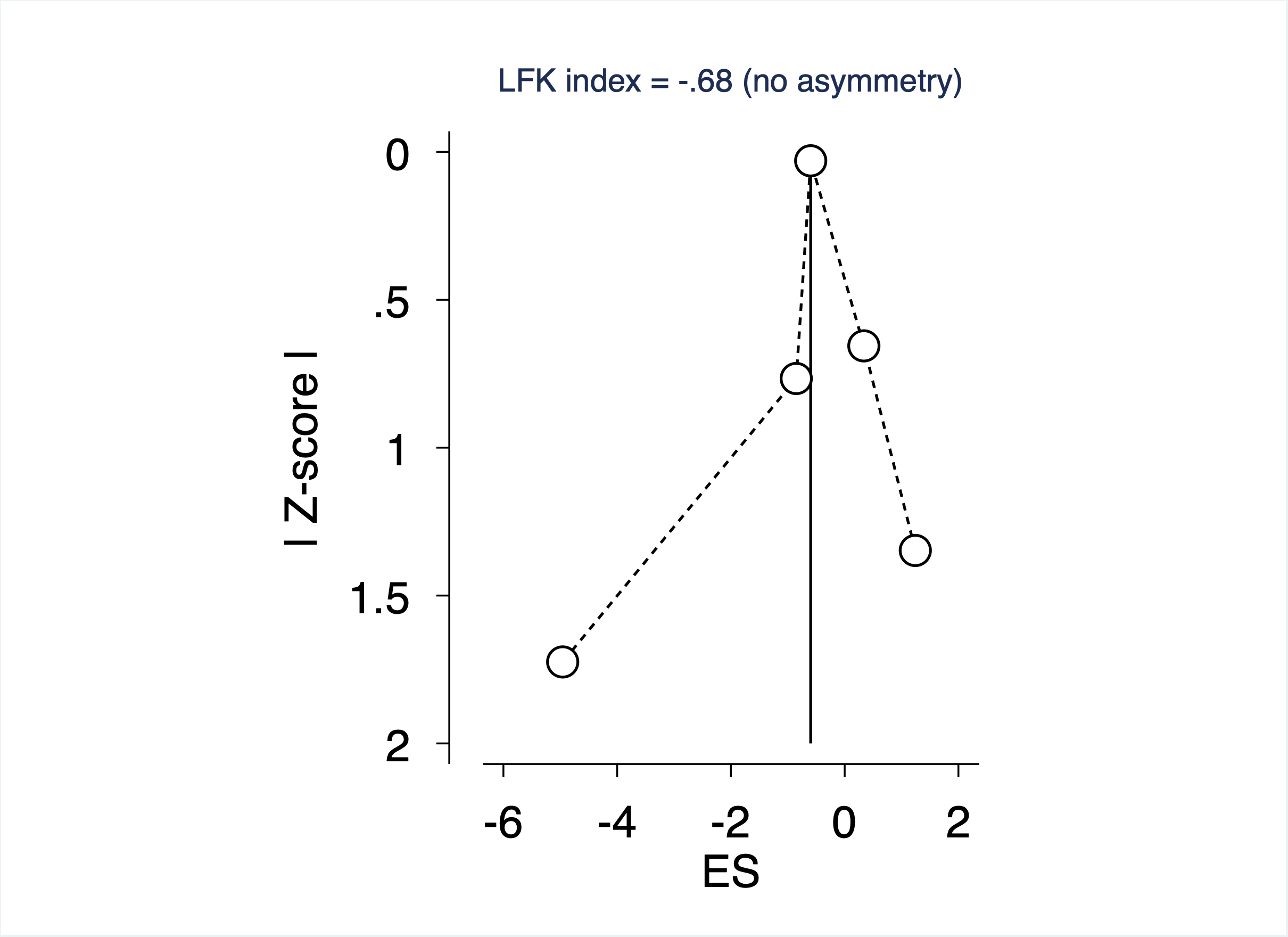

Supplement: Supplemental Information 4 [file peerj-13-19639-s004.tif]

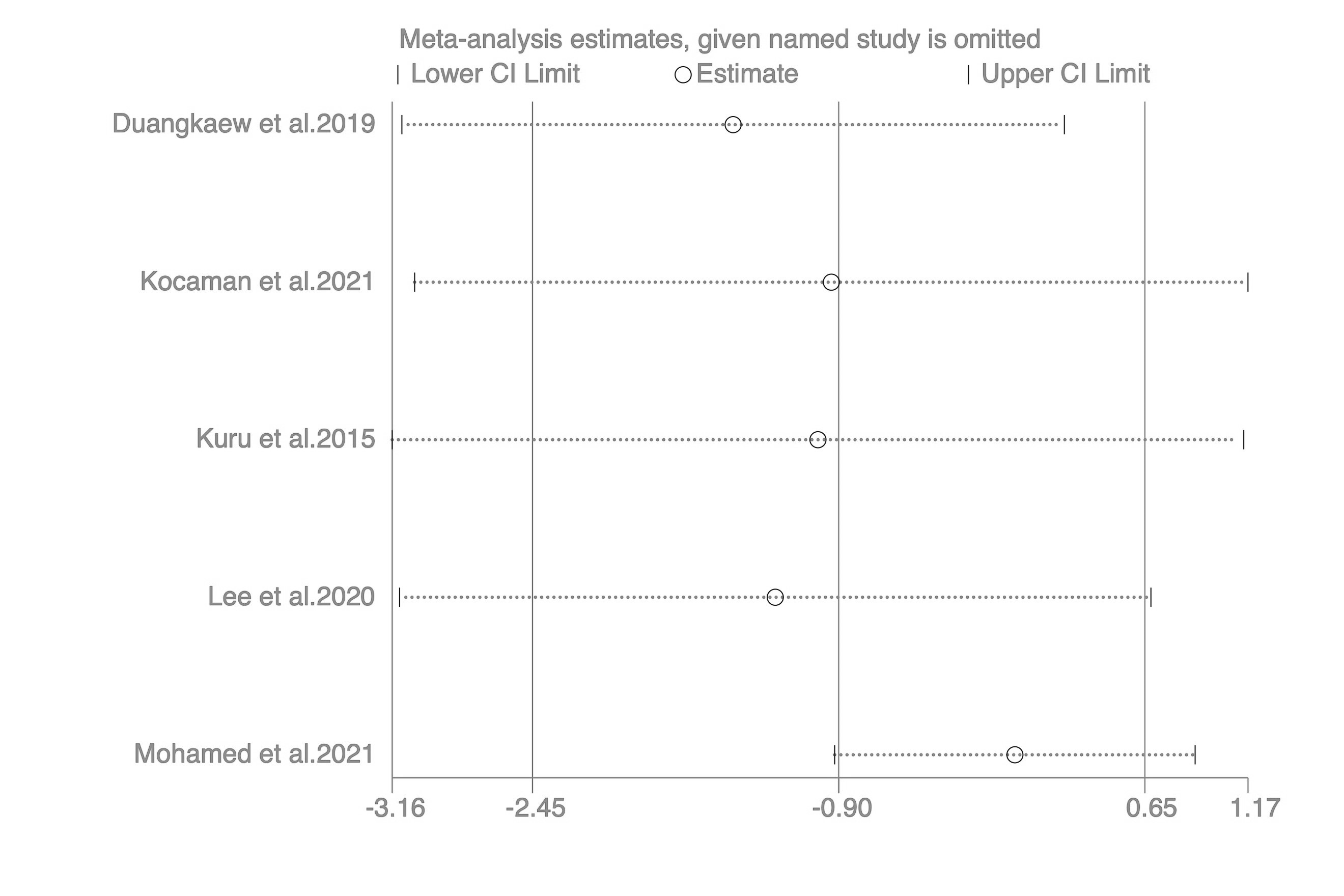

Supplement: Supplemental Information 5 [file peerj-13-19639-s005.tif]

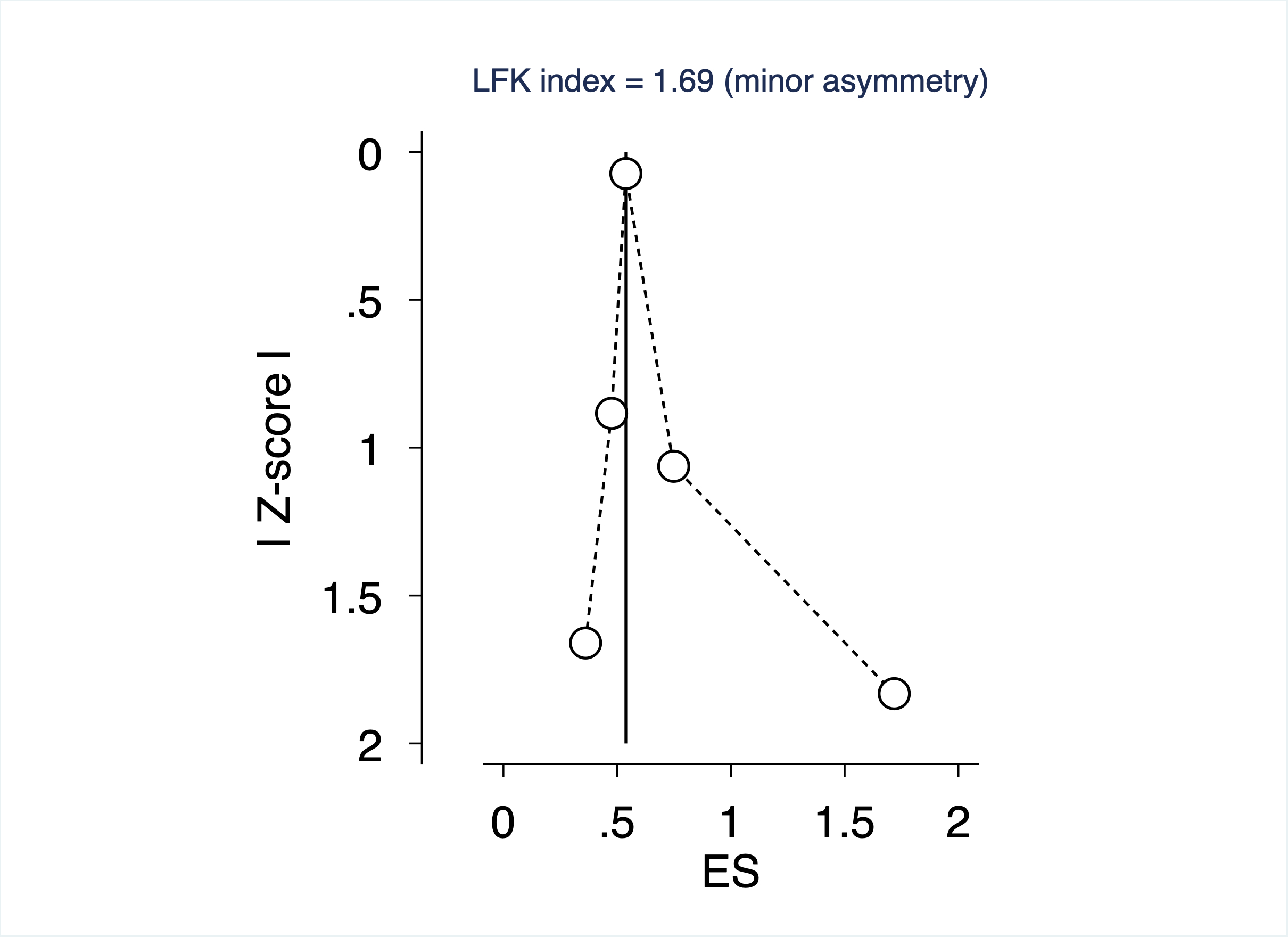

Supplement: Supplemental Information 6 [file peerj-13-19639-s006.tif]

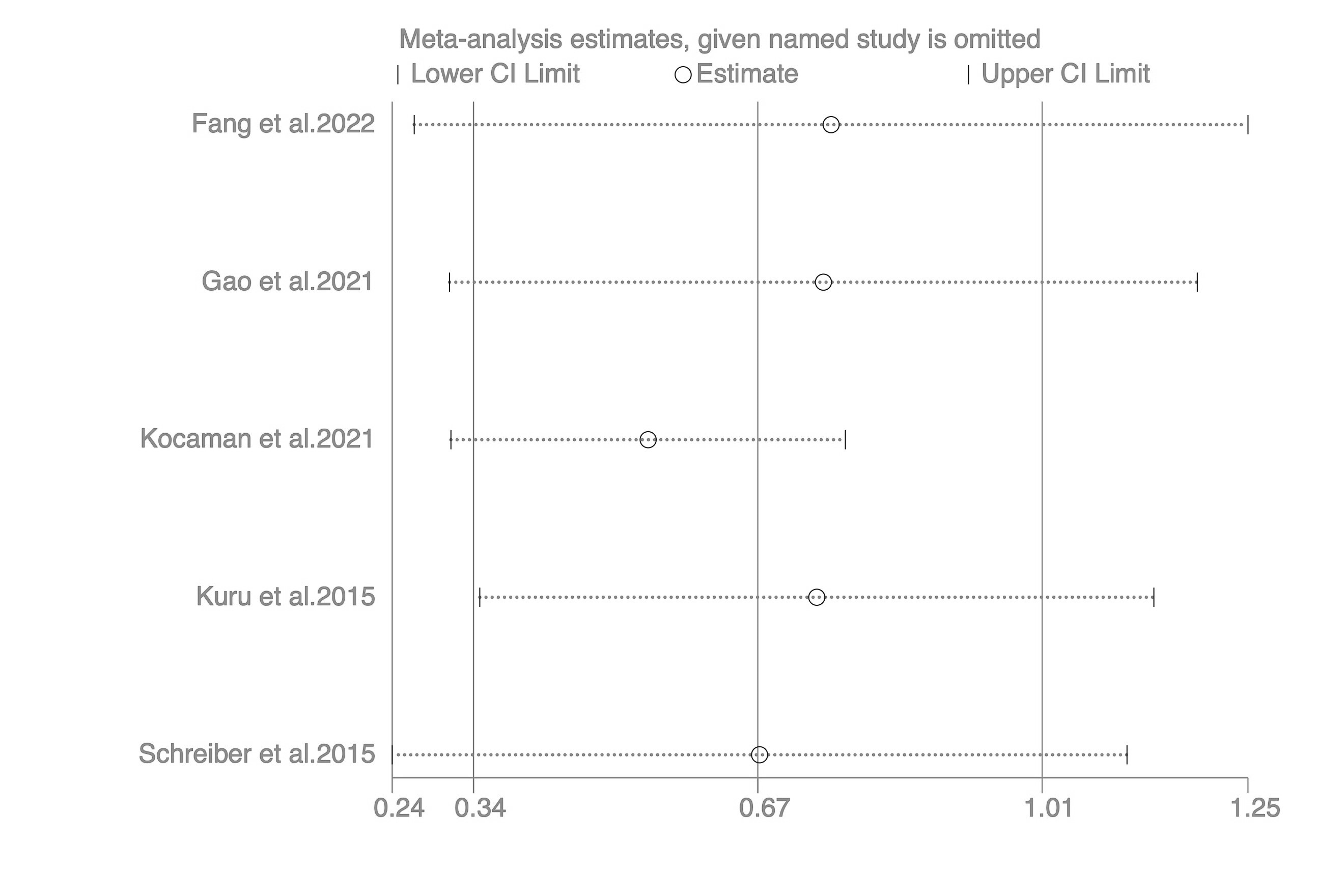

Supplement: Supplemental Information 7 [file peerj-13-19639-s007.tif]
