# Supplementary material for: Effectiveness of Schroth exercises for adolescent idiopathic scoliosis: a meta-analysis
Source: PeerJ. 2025 Jul 8;13:e19639. doi: 10.7717/peerj.19639 (PMC12248227; doi:10.7717/peerj.19639)
Supplement: Supplemental Information 8 [file peerj-13-19639-s008.docx]

**Supplementary Table 1: Detailed intervention protocols in the include studies (N=11)**

| **Study Identifier** | **Different protocols and the specific Schroth schools** |
| --- | --- |
| Kim et al.2016 | Schroth exercises were performed three times a week for 12 weeks. Each 60-min session consisted of preparation (cat walking and breathing exercise: 10 min), stretching (stretching the chest part: 5 min), the main exercise (lying right click concave, lying aside static postural control training, sitting posture adjustment exercise, and muscle cylinder: 40 min), and wrap-up (moving the ribs: 5 min). The Schroth exercise was applied in accordance with the bending shape of each subject, along with three-dimensional Schroth rotational breathing. |
| Gao et al. 2021 | The Schroth exercises were performed according to the Barcelona Scoliosis Physical Therapy School protocol. Auto elongation, asymmetrical sagittal straightening, frontal plane correction, rotational angular breathing, and stabilization variations were the primary principles. Patients received breath-training exercise, muscle strength control exercise, body-shape correction exercise, and balancing capacity exercises |
| Kocaman et al.2021 | The Schroth exercises were demonstrated and supervised by a certified and experienced physiotherapist (H.K.). Patients were placed in an asymmetric position to maximize correction in trunk symmetry. The Schroth program includes exercises for rotational breathing, spinal elongation, de-flexion, stretching, de-rotation, and strengthening, and these exercises were per- formed to improve the curvature, muscle strength, and endurance of postural muscles. During the Schroth exercises, rice bags, foam blocks, a stool, and long sticks were used to adjust the posture and give passive support. |
| Kuru et al.2015 | Treatment regimens lasted for six weeks (18 sessions) as an outpatient or home program. The patients in the Schroth exercise group started their exercise program under physiotherapist supervision for 1.5 hours a day, three days per week. The Schroth exercises were performed in an asymmet- ric position to maximise correction to achieve trunk symmetry. These exercises include spinal elongation, de-rotation, de-flexion, stretching, strengthening and rotational breathing exercises to maintain vertebral alignment |
| Mohamed et al.2021 | The exercises were performed using the RAB. The patient was instructed to breathe deeply during the self-correction of the curve to maxi- mally expand the chest wall; during exhalation, the patient was asked to increase the activation by keeping all corrections. Each pro- gram was given for one hour with a 2-min rest period between each exercise, three days a week, for six successive months. |
| Fang et al.2022 | Exercise program under physiotherapist supervision for 1.5h a day, three days per week for six weeks, combined with a daily home exercise program lasting for 30–45min. SBP consists of passive and active postural auto-correction exercises done repeatedly. These exercises include spinal elongation, de-rotation, de-flexion, stretching, strengthening, and rotational breathing exercises to maintain vertebral alignment |
| Schreiber et al.2016 | The six-month supervised Schroth PSSE intervention included five one-hour long private sessions delivered during the first two weeks, followed by weekly one-hour long group classes combined with a 30–45 min daily home exercise program. Exercises with the corrective movements required, the targeted curve type, the level of passive support involved, whether static or dynamic, and the dosages recommended. A Schroth curve classification algorithm and algorithms to guide the exercise prescription and progression for each Schroth curve type were developed to standardize treatment and ensure reproducibility |
| Lee et al.2020 | Schroth therapeutic exercise were treated for 2 hours, twice a week, for 12 weeks under the observation of therapists. The Schroth exercise was designed to apply a total of 29 movements according to the patient’s angle and type, and the degree of difficulty that the patient can accept. |
| Duangkeaw et al.2019 | Three-dimension Schroth exercises were used in this study. The whole process was under the supervision of a physical therapist trained in the Schroth technique and who received a diploma from the Institute of Asklepios in Germany. The exercise program was designed to suit each individual participant and consisted of breathing exercises, stretching and adjusting the curve of the spine with exercises Training sessions were scheduled for 2 days per week, 2 h per day for 6 consecutive weeks (total twelve days). |
| Schreiber et al.2015 | The 6-months supervised Schroth exercise intervention included five initial 1-h long private training sessions delivered during the first two weeks after baseline, followed by weekly 1-h long group classes combined with a 30–45 min daily home exercise program. A Schroth curve type classification algorithm and algorithms to guide the exercise prescription and progression for each of the four Schroth curve types were developed for this trial to standardize exercise delivery |
| HwangBo et al.2016 | Warm up for 10 minutes with 3D breathing exercises and cat walking followed by 45 minutes of main exercise, shoulder counter traction in side lying, shoulder counter traction in prone, Shoulder counter traction with elastic band in sitting, muscle cylinder in sitting, and cool down for 5 minutes with moving ribs |

C – Control; I – Intervention; NR – Not reported; NA – Not applicable; RCT – randomized controlled trial; SD – Standard deviation; USA – United States of America
